# The association between basilar artery wall thickness and posterior circulation ischemic stroke: insights from high-resolution vascular wall imaging in subclinical atherosclerosis

**DOI:** 10.3389/fneur.2025.1575681

**Published:** 2025-05-30

**Authors:** Zhen Zhang, Lirong Yi, Jie Zhou, Xiaojing He, Mingjing Yuan, Bo Xiang

**Affiliations:** ^1^Department of Radiology, The Affiliated Yongchuan Hospital of Chongqing Medical University, Chongqing, China; ^2^Department of Radiology, The Second Affiliated Hospital of Chongqing Medical University, Chongqing, China

**Keywords:** high-resolution magnetic resonance vascular wall imaging, basilar artery, vessel wall thickness, atherosclerosis, subclinical atherosclerosis

## Abstract

Basilar atherosclerosis is a major cause of posterior circulation ischemic stroke and it can affect neurological function negatively. Little is known about subclinical atherosclerotic changes before plaque formation. However, imaging changes in atherosclerotic plaque rupture and thrombosis have been extensively studied, which are major factors leading to the development of stroke. This study included 51 patients with posterior circulation ischemic stroke, 55 patients with high-risk factors for atherosclerosis, and 36 patients without high-risk factors for atherosclerosis. Based on high-resolution vascular wall imaging (HR-VWI) data, we quantitatively analyzed the differences in morphological abnormalities of the basilar artery wall between the three groups, including wall thickness, wall area, vessel area, and lumen area. This study aims to investigate the impact of abnormal basilar artery wall morphology, particularly on arterial wall thickness, on posterior circulation stroke development and its potential utility as a predictive biomarker. The results revealed that the stroke group exhibited significantly higher rates of cardiovascular risk factors (diabetes, hypertension, and elevated LDL-C) and increased basilar artery wall thickness compared to the high-risk group (*p* < 0.05). Logistic regression identified previous stroke, diabetes, and increased wall thickness as independent stroke risk factors. ROC analysis revealed strong associations between posterior circulation ischemic stroke and both vessel wall thickness alone (AUC = 0.806) and its combination with clinical features (AUC = 0.841), indicating potential diagnostic utility. These findings highlight the association between basilar artery wall morphology and stroke, supporting its potential role in risk stratification.

## 1 Introduction

Intracranial atherosclerosis is a leading cause of ischemic stroke, contributing to 30–50% of cases in Asian populations (1). It precipitates stroke through multiple mechanisms, including plaque formation, arterial stenosis, plaque instability, and hemodynamic alterations (2). These pathological changes can result in cerebral hypoperfusion, thromboembolism, or complete vessel occlusion, ultimately leading to ischemic brain injury (3). While high-resolution vessel wall imaging (HR-VWI) has advanced our understanding of plaque rupture in acute stroke (4–6), less is known about earlier, subclinical atherosclerotic changes preceding plaque formation.

Subclinical atherosclerosis is the pathophysiological stage characterized by pathological intimal thickening - the accumulation of lipids, inflammatory cells, and extracellular matrix in the arterial wall before visible plaque develops (7, 8). Studies have found that if the patient is treated with a small carotid intima-media thickness, plaque growth can be slowed or stopped (9, 10). Therefore, evaluating of subclinical atherosclerotic intima thickness can suggest early intervention, slow down or even reverse the atherosclerotic process, and reduce the risk of future disease and poor prognosis. Atherosclerosis progression differs substantially between intracranial and extracranial arteries. While extracranial disease progresses linearly, intracranial atherosclerosis develops later and shows distinct patterns (11), necessitating direct evaluation of intracranial vessels.

However, most existing studies have focused on changes in the intima thickness of the large blood vessels in the neck and the factors influencing these changes, emphasizing the correlation between the increase in extracranial blood vessel wall thickness and the formation of atherosclerotic plaque (12). Quantitative analysis of intracranial artery wall thickness based on HR-VWI has only been mentioned in a few assessments of non-atherosclerotic diseases (13, 14), such as vasculitis, and the long-term blood transfusion population. Posterior circulation strokes, though representing only 20–30% of ischemic strokes (1, 15), carry higher morbidity and mortality than anterior circulation events. Basilar artery (BA) occlusion accounts for approximately 1% of all ischemic strokes and about 10% of strokes due to an intracranial large vessel occlusion (16, 17). Although this condition may be relatively uncommon, it leads to potentially devastating consequences, including death or lock-in syndrome (18). Therefore, we selected BA as the object of analysis, aiming to study whether there is a difference in BA wall thickness between healthy people and patients with high-risk factors for atherosclerosis. Using our findings, we hope to guide early intervention in this group of people, ultimately preventing the occurrence and development of atherosclerotic post-circulation ischemic stroke.

## 2 Materials and methods

### 2.1 Image acquisition and processing

Instruments and parameters were set by the Vero 3.0 T MRI scanner (Siemens, Munich, Germany) with a 32-channel head and neck coil. The patient was positioned supine, and whole-brain HR-VWI was performed. The imaging protocol included conventional T1WI, T2WI, and coronal T2-FLAIR sequences, and diffusion-weighted imaging (DWI). Additionally, HR-VWI sequences, utilizing the T1-weighted sampling perfection with application-optimized contrasts using different flip angle evolution (T1-SPACE) and T2-weighted SPACE (T2-SPACE) sequences, were obtained by three-dimensional time-leap scanning, followed by axial scanning. HR-VWI scanning parameters included the following: TR, 800 ms; TE, 20 ms; slice thickness, 0.70 mm; voxel size, 0.70 × 0.70 × 0.70 (for T1-SAPCE) and TR, 1000 ms; TE, 81 ms; slice thickness, 0.80 mm; and voxel size, 0.80 × 0.80 × 0.80 mm (for T2-SPACE).

All image data were mapped using a commercial high-resolution magnetic resonance vessel wall analysis software (Vessel Explorer 2; TSImaging Healthcare, Beijing, China), and the mapping results were calculated automatically. The results included the area of the vascular lumen, the area of the lumen wall, the area of blood vessels, and the thickness of the blood vessel wall.

**Figure 1:**
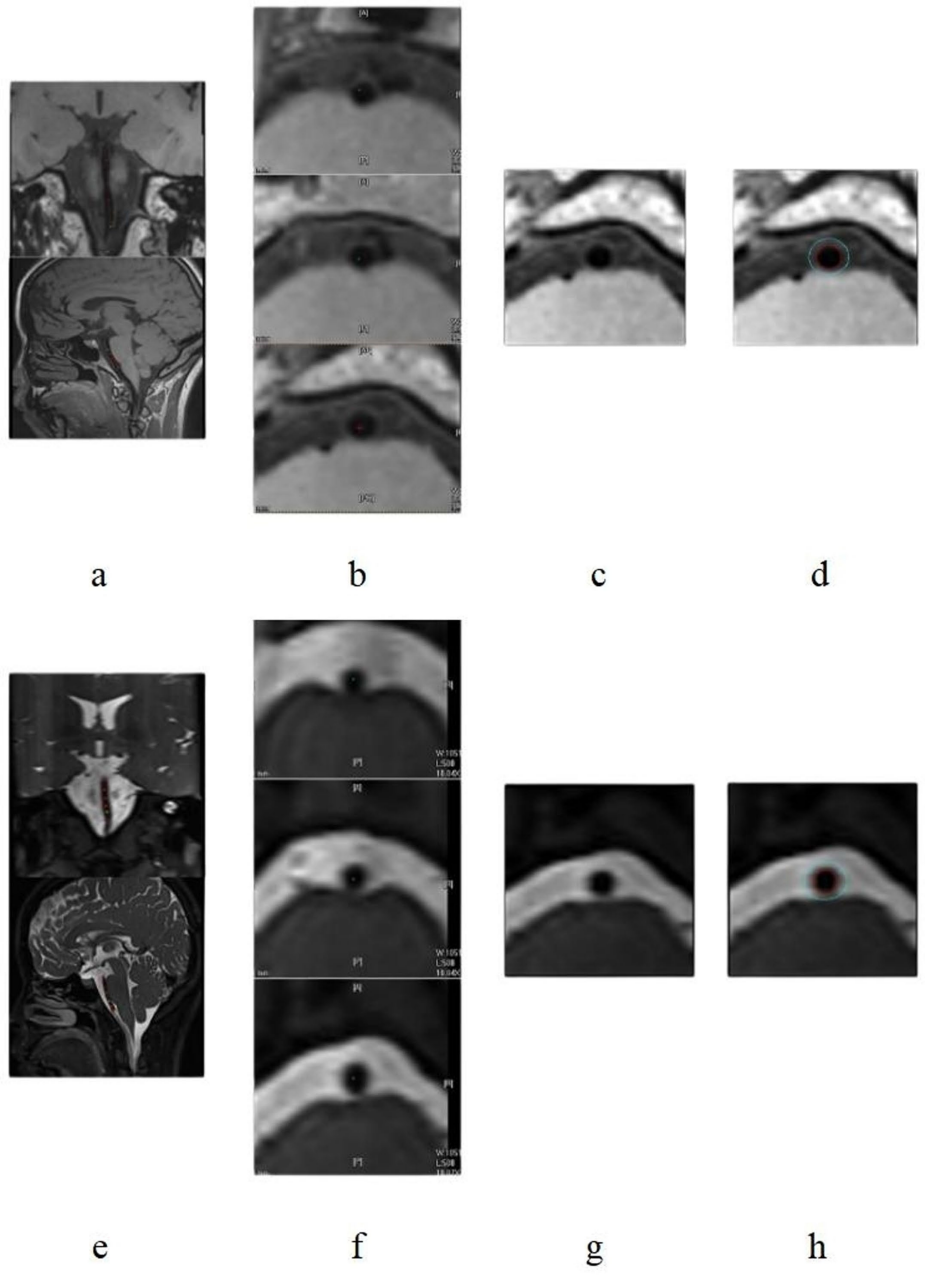
T1-SPACE and T2-SPACE images of a female patient aged 26 years from the control group. Parts **(a–d)** are T1-SPACE images; Parts **(e–h)** are T2-SPACE images. Images show the process of centerline extraction and vascular delineation.

**Figure 2:**
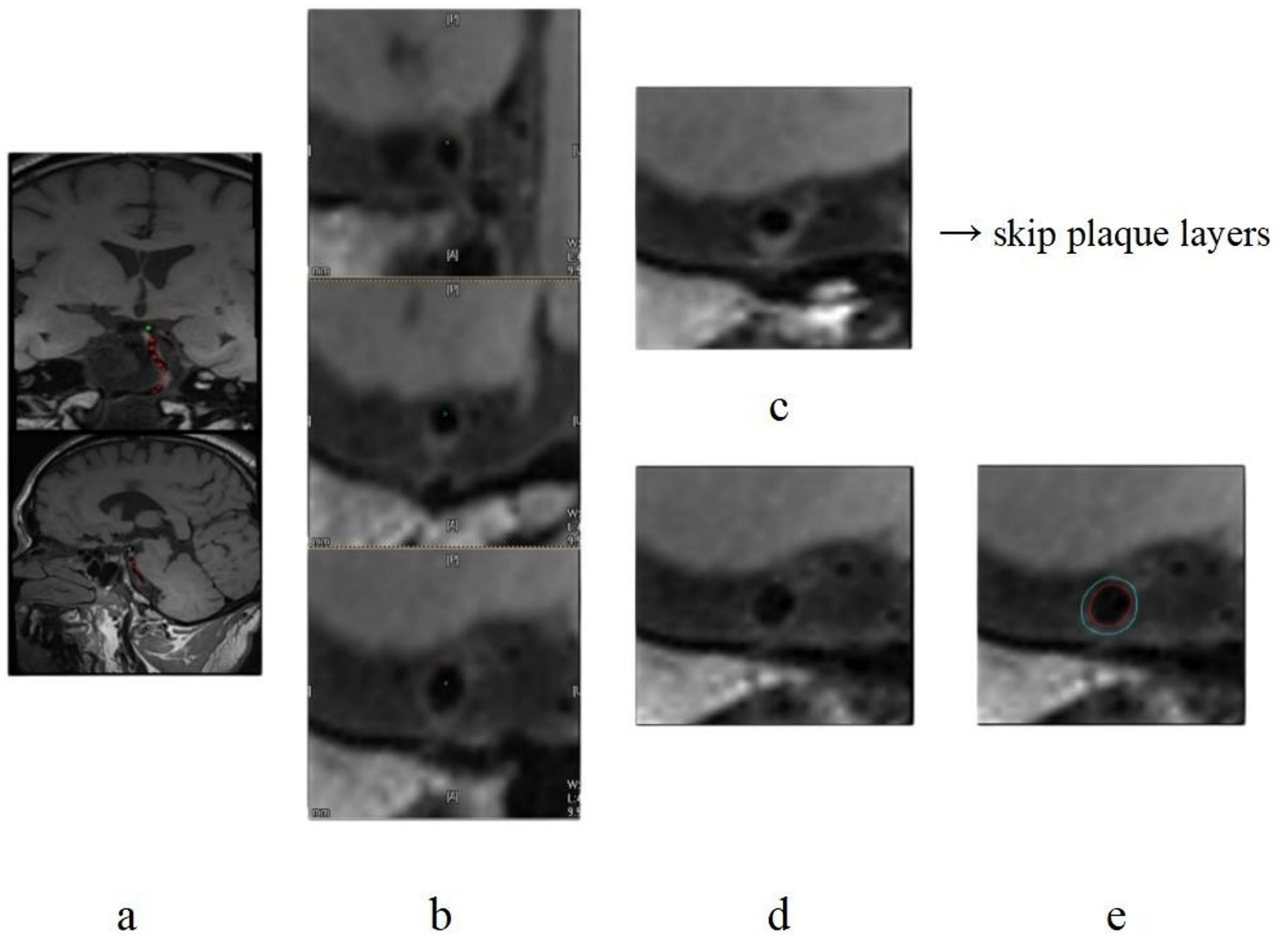
T1-SPACE images of a male patient aged 50 years from the stroke group. Parts **(a–e)** are T1-SPACE images. Images show the process of centerline extraction and the interface of vessel delineation.

Two radiologists independently reconstructed the BA along the vascular centerline to obtain axial images perpendicular to the lumen wall. The layers of reconstructed images were both 1 mm thick, and 10 layers of BA images were continuously outlined after the initial outlining of the confluent bilateral vertebral arteries. If this layer was the basal artery branch level in the sketch, it was skipped because of the irregular lumen; the layer of atherosclerotic plaque in the plaque group was not sketched; instead, continuously sketched the relatively normal vessel walls of upper and lower layers of the plaque (Figures 1, 2). The results of demographic, angiographic, and other physician measurements were double-anonymized.

### 2.2 Study design and subjects

This retrospective study analyzed 170 consecutive patients who underwent HR-VWI at Yongchuan Hospital of Chongqing Medical University between April 2021 and September 2023.

Inclusion criteria: (1) presence of suspected acute stroke symptoms (headache, dizziness, vomiting, limb weakness/numbness, speech impairment, or visual disturbances); (2) completion of both cranial DWI and HR-VWI examinations within 1 week post-admission; (3) Availability of complete clinical data.

Exclusion criteria: (1) diagnosis of other cerebrovascular diseases (Moyamoya disease, hemorrhagic stroke, or arterial dissection); (2) poor image quality precluding accurate boundary delineation.

**Figure 3:**
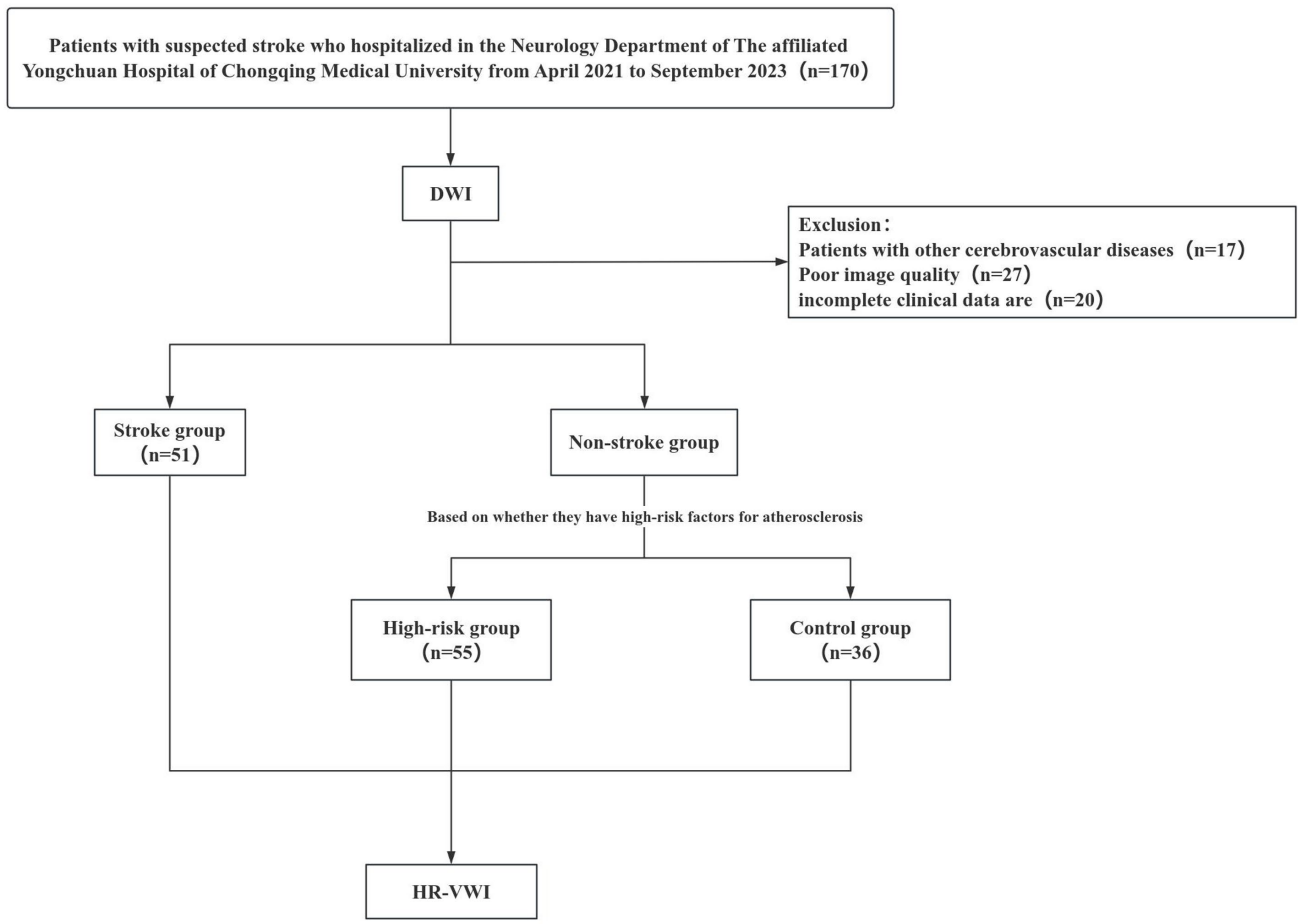
Flowchart of patients selection.

After excluding 47 patients for incomplete data or inadequate image quality and 17 for other cerebrovascular diseases, the final cohort comprised 106 patients, subdivided into a high-risk group (*n* = 55) and stroke group (*n* = 51). The study also included 36 healthy controls with normal clinical, imaging and laboratory findings for comparison. The high-risk factor group was defined as patients who met at least two of the following atherosclerotic risk factors based on laboratory tests and clinical evaluation: (1) hypertension (blood pressure ≥ 140/90 mmHg or use of antihypertensive medications); (2) diabetes mellitus (fasting glucose ≥ 7.0 mmol/L or hemoglobin A1c ≥ 6.5% or use of glucose-lowering medications); (3) hyperlipidemia (low-density lipoprotein cholesterol ≥ 3.4 mmol/L or use of lipid-lowering therapy); (4) current smoking status; (5) coronary artery disease history; and/or (6) previous stroke history. The stroke group was defined as patients who had posterior circulation infarction confirmed by DWI, characterized by high signal intensity on DWI. Collected clinical data encompassed smoking history, alcohol consumption, medical history (coronary heart disease, hypertension, diabetes, previous stroke), and lipid profiles. The institutional review board approved the study protocol, and all participants provided written informed consent (Figure 3).

### 2.3 Statistical analysis

**Figure 4:**
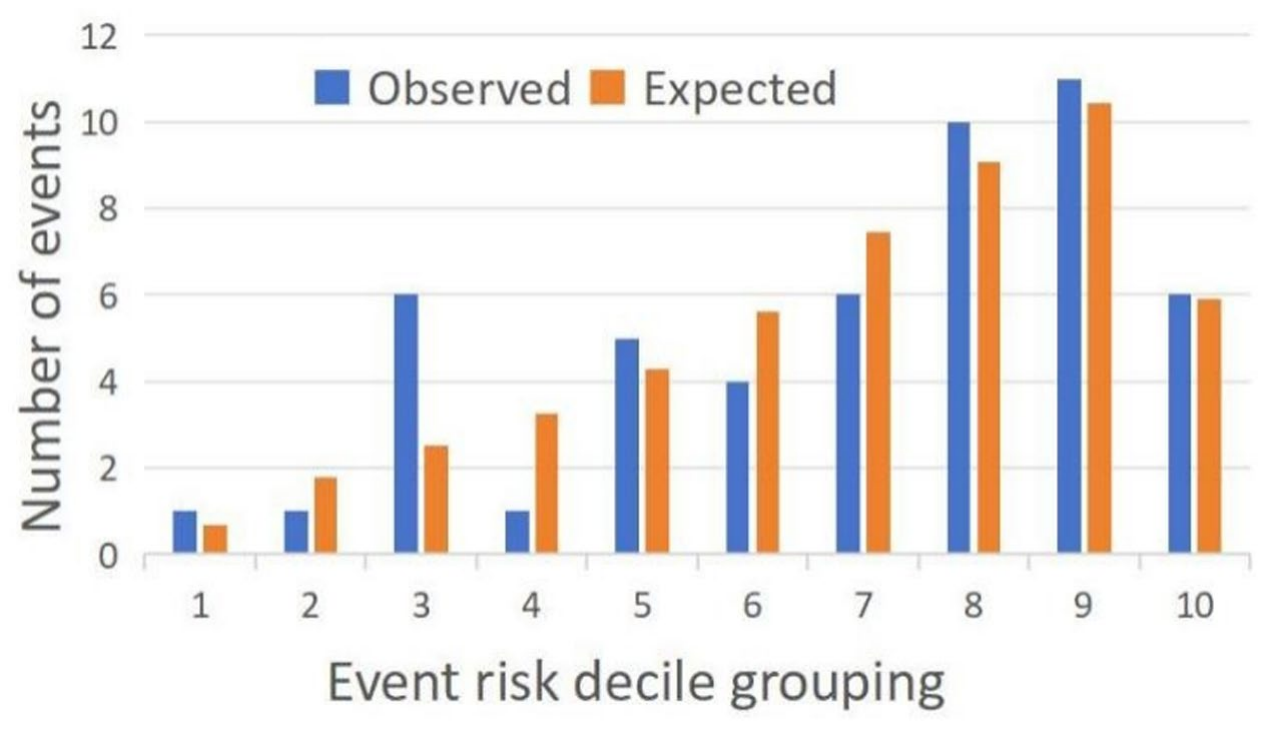
Hosmer–Lemeshow test model calibration histogram.

SPSS 25.0 statistical software (IBM Corporation, Armonk, NY, USA) was used to analyze the data. Depending on data distribution, continuous variables are presented as the means ± SD or medians (Q1, Q3), while categorical variables are expressed as counts and percentages. Categorical variables were analyzed with the *χ*^2^ test or Fisher’s exact test. Continuous variables were analyzed with ANOVA or the Kruskal-Wallis test, and pairwise comparisons were conducted between groups. Variables identified as statistically significant (*p* < 0.05) in the univariate analysis were subsequently included in a binary logistic regression model to further assess their association with the outcome. In addition, the correlation between vascular wall indices and age in the control group was examined by Pearson’s correlation test. A two-tailed *p* < 0.05 was considered indicative of statistical significance. The discriminative performance of vascular wall indicators and their combination with clinical indicators for posterior circulation ischemic stroke was assessed using the area under the receiver operating characteristic (ROC) curve (AUC), with model calibration evaluated by the Hosmer-Lemeshow test (Figure 4).

## 3 Results

### 3.1 Difference analysis of clinical data and laboratory indicators

Based on the standardized HR-VWI acquisition and measurement protocols described in the Methods section, the study population comprised 142 participants divided into control (*n* = 36), high-risk (*n* = 55), and stroke (*n* = 51) groups. As shown in Table 1, demographic analysis revealed comparable age (*F* = 2.217, *p* = 0.113) and sex distribution (*χ*^2^ = 0.040, *p* = 0.980) across groups. The stroke group demonstrated significantly higher prevalence of stroke history (33.3% vs. 9.1%, *χ*^2^ = 9.456, *p* = 0.002), coronary heart disease (22.0% vs. 5.5%, *χ*^2^ = 6.205, *p* = 0.013), hypertension (70.6% vs. 47.3%, *χ*^2^ = 5.925, *p* = 0.015), and diabetes (37.3% vs. 20.0%, *χ*^2^ = 3.883, *p* = 0.049) compared to the high-risk group. Lipid analysis showed significantly elevated triglycerides (35.3% vs. 18.2%, *χ*^2^ = 3.987, *p* = 0.046) and low-density lipoprotein cholesterol (LDL-C) levels (58.8% vs. 36.4%, *χ*^2^ = 5.357, *p* = 0.021) in stroke patients, while total cholesterol (*p* = 0.881) and high-density lipoprotein cholesterol (HDL-C) (*p* = 0.183) showed no significant differences. Smoking (*p* = 0.656) and alcohol consumption (*p* = 0.500) histories were comparable between stroke and high-risk groups. These findings demonstrate significant associations between vascular comorbidities/dyslipidemia and stroke occurrence, underscoring the importance of monitoring these factors in high-risk populations.

**Table 1:** Differential analysis of clinical data.

Variable	Control group (*n* = 36)	High-risk factor group (*n* = 55)	Stroke group (*n* = 51)	*F*/X^2^	*p*
Age (years) *	53.81 ± 15.51	54.87 ± 13.68	56.69 ± 14.38	2.217	0.113
Sex (male)	13 (36.1)	21 (38.2)	19 (37.3)	0.040	0.980
Smoking history	—	12 (21.8)	13 (25.5)	0.198	0.656
Drinking history	—	11 (20.0)	13 (25.5)	0.455	0.500
Stroke history	—	5 (9.1)	17 (33.3)	9.456	0.002
Coronary heart disease	—	3 (5.5)	11 (22.0)	6.205	0.013
Hypertension	—	26 (47.3)	35 (70.6)	5.925	0.015
Diabetes	—	11 (20.0)	19 (37.3)	3.883	0.049
High level of total cholesterol	—	37 (67.3)	35 (68.6)	0.022	0.881
High level of triglycerides	—	10 (18.2)	18 (35.3)	3.987	0.046
High level of low-density lipoprotein	—	20 (36.4)	30 (58.8)	5.357	0.021
High level of high-density lipoprotein	—	32 (58.2)	36 (70.6)	1.771	0.183

*Data is mean ± SD.

### 3.2 Difference analysis of calculated indices for vascular wall parameters

Consistent with the image processing methodology, wall thickness showed progressive increases from control (0.90 ± 0.09 mm) to high-risk (1.08 ± 0.09 mm) to stroke groups (1.22 ± 0.14 mm), with all inter-group comparisons being statistically significant (*p* < 0.001). For wall area, both the high-risk group (median = 14.63 mm^2^, IQR = 12.99–17.00) and stroke group (16.74 ± 4.99 mm^2^) demonstrated significantly larger values than the control group (12.38 ± 3.21 mm^2^, *p* < 0.008), though no significant difference existed between the two patient groups (*p* > 0.05). Vessel area was significantly larger in the stroke group (26.31 ± 8.11 mm^2^) compared to controls (22.08 ± 6.74 mm^2^, *p* = 0.029), while the high-risk group (median = 23.57 mm^2^, IQR = 19.78–26.67) showed intermediate values. Lumen area remained comparable across all groups (Table 2). The observed gradual thickening of arterial walls across the risk spectrum, particularly without corresponding lumen changes, suggests that vascular remodeling occurs early in the disease process. These findings support the potential clinical utility of wall thickness measurement as a sensitive marker for vascular health assessment in at-risk populations.

**Table 2:** Differential analysis of vascular wall indices.

Variable	Control group (*n* = 36)	High-risk factor group (*n* = 55)	Stroke group (*n* = 51)	H/X^2^	*p*
Wall thickness (mm)	0.90 ± 0.09*	1.08 ± 0.09*	1.22 ± 0.14*	84.328	< 0.001 ^abc^
Wall area (mm^2^)	12.38 ± 3.21*	14.63 (12.99–17.00)^♯^	16.74 ± 4.99*	20.881	< 0.008 ^ab^
Vessel area (mm^2^)	22.08 ± 6.74*	23.57 (19.78–26.67)^♯^	26.31 ± 8.11*	6.741	0.029 ^b^
Lumen area (mm^2^)	9.18 (7.21, 11.15)^♯^	8.51 (6.84–10.95)^♯^	8.95 (7.37–11.09)^♯^	0.892	0.649

^a^Control group vs. High-risk factor group.

^b^Control group vs. Stroke group.

^c^High-risk factor group vs. Stroke group.

*means ± SD.

^♯^medians (Q1, Q3).

### 3.3 Difference analysis of age for vascular wall parameters

**Figure 5:**
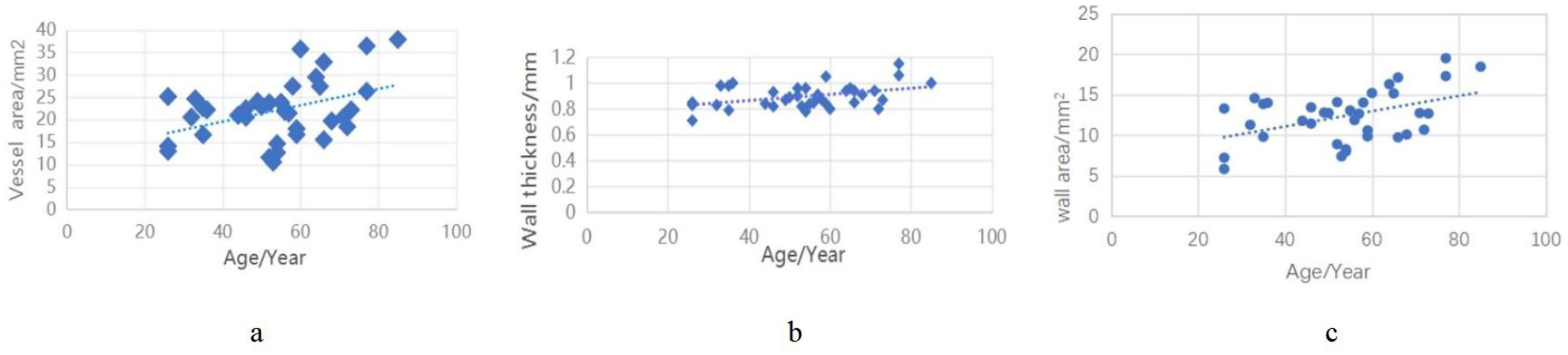
Correlation of vascular wall indices in the control group with age.

In the control group, we analyzed age-related correlations with vascular wall parameters. The results demonstrated significant positive correlations between age and all measured wall characteristics: wall thickness (*r* = 0.418, *p* < 0.01), wall area (*r* = 0.407, *p* < 0.01), and vessel area (*r* = 456, *p* < 0.05) (Figure 5).

### 3.4 Logistic regression analysis of stroke and high-risk factors

Binary logistic regression analysis with stroke occurrence as the dependent variable revealed that previous stroke history (*p* = 0.036), diabetes mellitus (*p* = 0.048), and increased wall thickness (*p* < 0.001) emerged as independent risk factors for stroke in the multivariable analysis. Although wall area demonstrated significance in univariable analysis (*t* = 3.26, *p* = 0.001), it failed to maintain independent predictive value when adjusted for other variables (*p* = 0.093) (Table 3).

**Table 3:** Binary logistic regression analysis of stroke and vascular wall indexes.

Variable	Univariable analysis		Multivariable analysis		
	*t*/*χ*^2^	*p* value	OR value	95% CI	*p* value
Stroke history	19.346	<0.001	4.338	1.097–17.151	0.036
Diabetes	11.094	0.001	3.029	1.011–9.075	0.048
Wall area (mm2)	3.260	0.001	0.889	0.776–1.020	0.093
Wall thickness (mm)	8.215	<0.001	780008.612	1302.933–466, 956,927.0	< 0.001

### 3.5 Evaluation for identification of posterior circulation ischemic stroke

**Figure 6:**
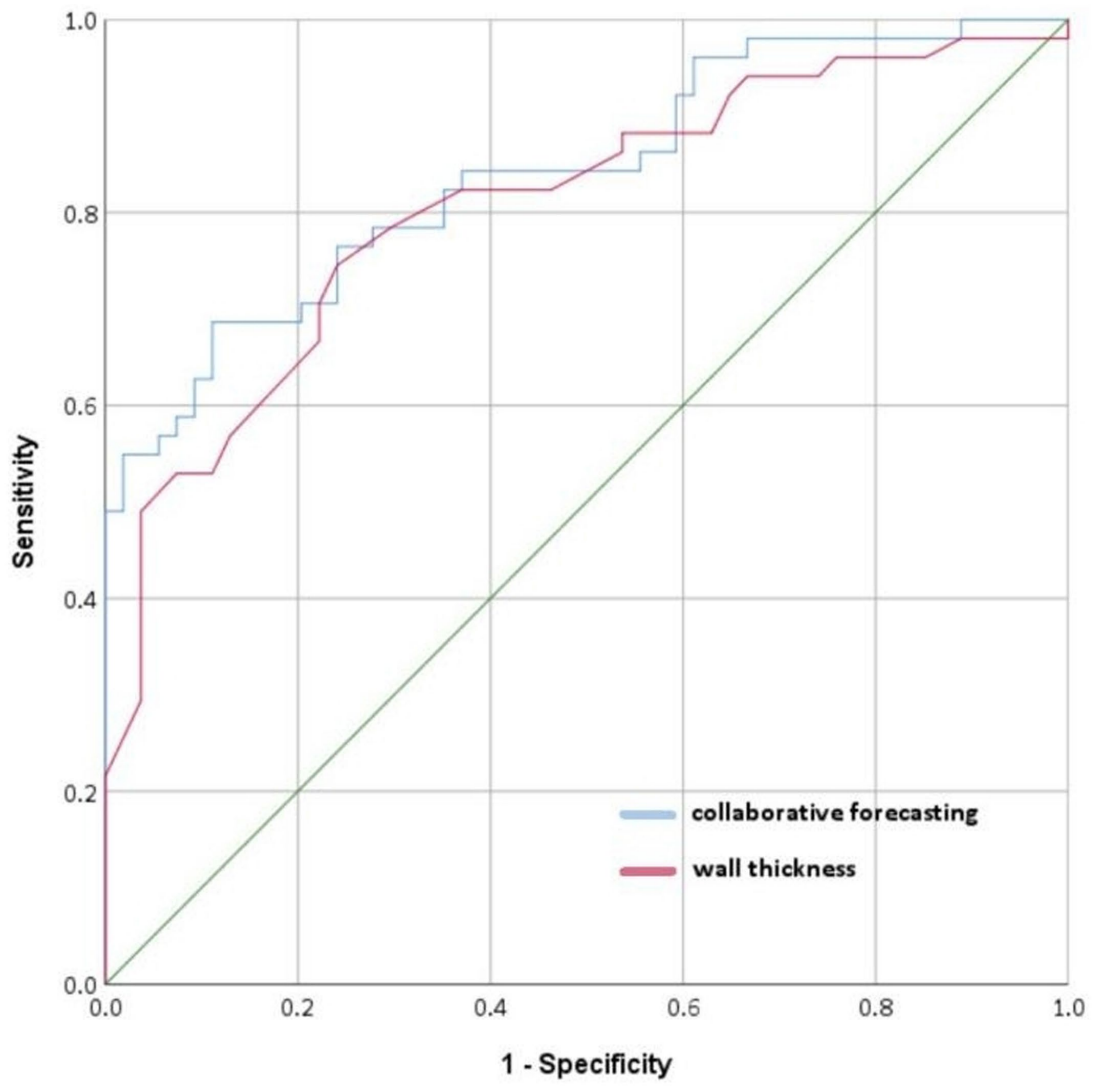
Basilar atherosclerosis assessment by ROCs.

Following the predefined statistical analysis plan, ROC analysis was performed to assess the discriminative performance of: (1) vessel wall thickness alone, and (2) vessel wall thickness combined with clinical features (including diabetes mellitus and stroke history) for post-cardiac surgery stroke (Figure 6). The AUC for identifying posterior circulation ischemic stroke based on vessel wall thickness was 0.806 (sensitivity 74.50%, specificity 75.90%). The combined model showed improved discriminative ability with an AUC of 0.841 (sensitivity 68.60%, specificity 88.90%) (Table 4). As shown in Figure 4, the Hosmer-Lemeshow test indicated good model calibration (*χ*^2^ = 12.347, *p* = 0.136). These findings support the potential clinical utility of this combined approach for stroke risk stratification, though external validation is warranted to confirm generalizability.

**Table 4:** Association between wall thickness measurements and basilar artery atherosclerosis severity in stroke patients.

Variable	AUC	95% CI	*p*	Sensitivity (%)	Specificity (%)	Youden index
Wall thickness	0.806	0.766–0.917	< 0.001	74.50	75.90	0.504
Collaborative forecasting	0.841	0.722–0.891	< 0.001	68.60	88.90	0.575

Collaborative forecasting: basilar artery wall thickness, history of stroke and history of diabetes were combined for analysis.

## 4 Discussion

HR-VWI, as a non-invasive magnetic resonance examination, has the advantage of high soft tissue resolution. Previous studies have found that HR-VWI has good repeatability in measuring BA wall thickness in healthy individuals, and the intra-class correlation coefficient range is 0.83–0.94, meaning it can meet the requirements for qualitative and quantitative assessment of intracranial vascular wall lesions (14, 19). The application of HR-VWI can improve the identification of basilar artery anomalies (20). Based on HR-VWI imaging, in the comparative analysis between the high-risk factor and stroke groups in this study, it was found that the lumen wall thickness in the stroke group was greater than that in the high-risk factor group. Additionally, there were statistically significant differences in the history of hypertension, diabetes, stroke, coronary heart disease, and hyperlipidemia in the stroke group (*p* < 0.05). This may be due to factors such as hypertension and hyperlipidemia that can induce dysregulation of lipid metabolism and inflammatory response in the body (6). According to the injury response hypothesis of the early onset of atherosclerosis, the essence of atherosclerosis emphasizes endothelial dysfunction, which is characterized by the reduced bioavailability of nitric oxide by vascular endothelium and the retention of cholesterol-carrying LDL-C under the combined action of these factors, resulting in the accumulation of extracellular matrix and endothelial activation. The above pathophysiological mechanisms further promote the development of atherosclerosis (21, 22). Cardiovascular risk factors affect specific segments of intracranial vessels in high-resolution vessel wall imaging (23). Previous studies of diabetic patients found that abnormal blood sugar levels can lead to thickening of the carotid intima (24). In experiments of mice fed a high-fructose, high-fat diet, it was observed that a high-fat diet resulted in an increase in the thickness of the BA wall of mice and that the leptin level increased with the increase in wall thickness (22). Since leptin is associated with hyperinsulinemia and glucose intolerance and has atherogenic effects (25), it is speculated that abnormal blood glucose in the intracranial arteries is also associated with lumen wall thickening. In our stroke group, the average thickness of the lumen wall was greater than that in the control and high-risk factor groups, and the proportion of diabetes patients was also higher than that in the high-risk factor group. These data indicate that the thickening of the lumen wall and the development of diabetes will increase the risk of stroke, but it cannot be confirmed that the increase in lumen wall thickness directly relates to blood glucose. The relationship between blood glucose and lumen wall thickness needs to be verified by single-factor analysis. There was no statistically significant difference in lumen area between the three groups. This result may be due to the large congenital difference in vessel diameter among different individuals. The narrow diameter and small lumen area do not necessarily suggest the existence of stenosis or atherosclerosis. This study confirms that diabetes mellitus, history of stroke, and vessel wall thickening are independent risk factors for stroke. However, the presence of vascular positive remodeling (characterized by increased wall thickness and vessel area without luminal stenosis) makes relying solely on wall thickness unreliable for risk assessment. Therefore, a multivariable approach incorporating multiple indicators is recommended to improve the accuracy of stroke risk prediction. The combination of these three factors demonstrates superior discriminative power compared to wall thickness alone, underscoring the importance of comprehensive risk stratification and the implementation of primary and secondary prevention strategies in patients with vessel wall thickening.

In addition, the correlation analysis of vascular wall indicators and age in the control group in this study found that lumen wall thickness, vessel area, and lumen wall area were positively correlated with age. This is consistent with the slight increase observed by Cogswell et al. in the scatterplot of BA thickness and age distribution in healthy screening populations. However, whether the increasing trend of intracranial artery wall thickness is an early-stage reaction of atherosclerotic changes or a physiological change during aging is still unclear and needs to be further clarified by screening and longitudinal follow-up of healthy people. The thickness and area of the BA wall in the high-risk factor and stroke groups were greater than those in the control group, and there was also a statistical difference in wall thickness between the high-risk factor and stroke groups. It was confirmed from imaging that atherosclerosis is a continuous development process. As the thickness of the artery wall increases, atherosclerosis changes from simple vessel wall thickening to plaque formation, which increases the risk of stroke. In non-atherosclerotic diseases, vascular reactivity has been demonstrated to decrease with thickening of the intracranial artery walls (14). The increase in vascular area in the stroke group compared to the control group may reflect positive remodeling of the BA wall under the combined effects of high-risk atherosclerotic factors, leading to an overall expansion of the vessel wall area. This expansion correlates with arterial wall thickness.

Several important limitations of this study must be acknowledged. First, as a single-center retrospective study, certain clinically relevant atherosclerosis risk factors, including body mass index and dietary patterns, were not systematically collected or analyzed. Furthermore, the absence of longitudinal follow-up data limits our ability to assess the predictive value of vascular wall thickening over time—a critical consideration for future research. Second, the control group comprised only 36 participants, raising concerns about selection bias and potentially limiting the generalizability of our findings to healthy populations. Third, we did not account for disease duration or severity stratification among cases, which precludes analysis of how chronicity or metabolic control of conditions such as hypertension and diabetes may influence vascular remodeling. Fourth, the logistic regression analysis did not adjust for potential confounders including age, sex, hypertension, and cholesterol levels, which may affect the interpretation of identified risk factors. Fifth, while we focused on early subclinical atherosclerosis through wall thickness measurements, the lack of systematic plaque characterization (presence/absence, morphology, and composition) prevents direct assessment of the relationship between pre-plaque changes and established atherosclerotic lesions. Sixth, our study did not distinguish degrees of wall thickening, instead relying on binary classification, which may overlook clinically meaningful variations in vascular pathology. Finally, technical constraints must be considered: accurate wall thickness measurement requires the vessel wall to be at least twice the voxel size (26). Given the small diameter of the basilar artery, measurement precision may be compromised, potentially obscuring subtle pathological changes. Advances in high-resolution MRI technology are needed to improve spatial resolution in future studies. We further recognize that our imaging-based approach cannot determine the underlying etiology of wall thickening—whether inflammatory, lipid-driven, or hypertrophic—without histopathological correlation. This represents a key limitation in interpreting the biological significance of our findings.

In conclusion, the BA wall thickness is greater in people at high risk of atherosclerosis than in normal healthy people. BA wall thickening combined with atherosclerosis risk factors is more likely to lead to atherosclerotic-related stroke. Therefore, at the level of subclinical atherosclerosis, the graded management of lumen wall thickness in high-risk populations before plaque formation can prevent intracranial plaque formation to a certain extent to achieve the purpose of health maintenance.

## Data Availability

The raw data supporting the conclusions of this article will be made available by the authors, without undue reservation.
